# Supplementary material for: Complexome profiling of the Chlamydomonas psb28 mutant reveals TEF5 as an early PSII assembly factor
Source: Plant Cell. 2025 Mar 17;37(6):koaf055. doi: 10.1093/plcell/koaf055 (PMC12164590; doi:10.1093/plcell/koaf055)
Supplement: koaf055_Supplementary_Data [file koaf055_supplementary_data.zip › psb28_interactive.zip!/plots/intensity/qualityPlots/peptides/TEF5_Cre09.g411200.t1.2.html]

### Cre09.g411200.t1.2

Trivial name: TEF5  
SeqCoverage [median]: 21.60  
Mass: 33.17 kDa  
  
 

| Sequence | MissedCleavage | Mass [Da] | wtIntensity | muIntensity | Score |
| --- | --- | --- | --- | --- | --- |
| DGSIQAWYPNNPVLR | 0 | 1728.86 | 0.00 | 986.56 | 64.44 |
| EVEVDGR | 0 | 802.38 | 2583.18 | 13555.85 | 148.98 |
| KEVEVDGR | 1 | 930.48 | 460.93 | 0.00 | 69.81 |
| LAQEAIYVDVSDSR | 0 | 1564.77 | 4024.78 | 23654.00 | 194.24 |
| NQIYAIEAR | 0 | 1076.56 | 19907.18 | 35546.28 | 166.52 |
| SPAEGAYSEGFIK | 0 | 1354.64 | 24381.35 | 61718.74 | 281.41 |
| YNNAVTR | 0 | 836.41 | 11906.67 | 35941.25 | 170.87 |
|  |  |  | 63264.1 | 171402.7 |  |
